# Amentoflavone Inhibits HSV-1 and ACV-Resistant Strain Infection by Suppressing Viral Early Infection

**DOI:** 10.3390/v11050466

**Published:** 2019-05-22

**Authors:** Feng Li, Xiaowei Song, Guifeng Su, Yiliang Wang, Zhaoyang Wang, Jiaoyan Jia, Shurong Qing, Lianzhou Huang, Yuan Wang, Kai Zheng, Yifei Wang

**Affiliations:** 1 Institute of Biomedicine, College of Life Science and Technology, Jinan University, Guangzhou 510632, China; li_fengfeng@163.com (F.L.); xiaowei@stu2017.jnu.edu.cn (X.S.); qq154931686@outlook.com (G.S.); yiliang_wang@foxmail.com (Y.W.); wzy1109@stu2017.jnu.edu.cn (Z.W.); jjy1017@stu2017.jnu.edu.cn (J.J.); qsr1260132236@gmail.com (S.Q.); 13535221869@163.com (L.H.); eleneaWy@163.com (Y.W.); 2 School of Pharmaceutical Sciences, Health Science Center, Shenzhen University, Shenzhen 518060, China

**Keywords:** herpes simplex virus 1, ACV-resistant strains, amentoflavone, cofilin, F-actin dynamic

## Abstract

Infection of Herpes simplex virus 1 (HSV-1) induces severe clinical disorders, such as herpes simplex encephalitis and keratitis. Acyclovir (ACV) is the current therapeutic drug against viral infection and ACV-resistant strains have gradually emerged, leading to the requirement for novel antiviral agents. In this study, we exhibited the antiviral activity of amentoflavone, a naturally occurring biflavonoid, toward HSV-1 and ACV-resistant strains. Amentoflavone significantly inhibited infection of HSV-1 (F strain), as well as several ACV-resistant strains including HSV-1/106, HSV-1/153 and HSV-1/Blue at high concentrations. Time-of-drug-addition assay further revealed that amentoflavone mainly impaired HSV-1 early infection. More detailed study demonstrated that amentoflavone affected cofilin-mediated F-actin reorganization and reduced the intracellular transportation of HSV-1 from the cell membrane to the nucleus. In addition, amentoflavone substantially decreased transcription of viral immediate early genes. Collectively, amentoflavone showed strong antiviral activity against HSV-1 and ACV-resistant strains, and amentoflavone could be a promising therapeutic candidate for HSV-1 pathogenesis.

## 1. Introduction

Herpes Simplex Virus type 1 (HSV-1) is a DNA virus with envelope and belongs to α subfamily *Herpesviridae* [1]. HSV-1 can cause a variety of clinical disorders, such as encephalitis and keratitis, and importantly, the mortality rate of HSV-1-induced encephalitis is about 70% [2]. HSV-1 establishes latent infection in peripheral nerve ganglia and the central nervous system, and the latent virus can be reactivated under psychological stress [3]. Further increasing evidences indicate that HSV-1 infection plays a role in the development of neurodegeneration disease, such Alzheimer’s disease [4]. Currently, the clinically therapeutic drug against HSV-1 infection is Acyclovir (ACV), a nucleoside analog that has been hailed as a milestone in the history of antiviral drugs [5]. However, ACV-resistant viruses appear gradually [6], making developing novel and high-efficiency antiviral agents important. 

Cell cytoskeleton, especially actin filaments and microtubules, plays critical functions in various cellular activities, such as cell motility and cell division, as well as pathogen infection [7,8]. Every stage of virus life cycle, from entry to egress, is tightly associated with the reorganization of cell cytoskeleton [9,10]. Cofilin is a key regulator in controlling the temporal and spatial extent of actin dynamics and different viruses have evolved various molecular mechanisms to manipulate cofilin activity to subvert the actin cytoskeletal system in host cells, promoting their internalization into the target cells and facilitating their intracellular and intercellular dissemination [11,12]. For instance, we have demonstrated previously that HSV-1 entry and penetration induces the rearrangement of F-actin as well as the initial inactivation and subsequent activation of cofilin [13]. Upon viral binding, EGFR-PI3K signaling was activated to induce cofilin phosphorylation and F-actin polymerization, which in turn promoted HSV-1’s efficient entry. Subsequent viral penetration activated cofilin (dephosphorylation of cofilin), leading to the fragmentation of existing actin filaments, and presumably, loosening of the actin cortex and facilitation of virus trafficking [14]. Therefore, interruption of cofilin-mediated dynamic actin regulation represents a promising antiviral strategy.

**Figure 1:**
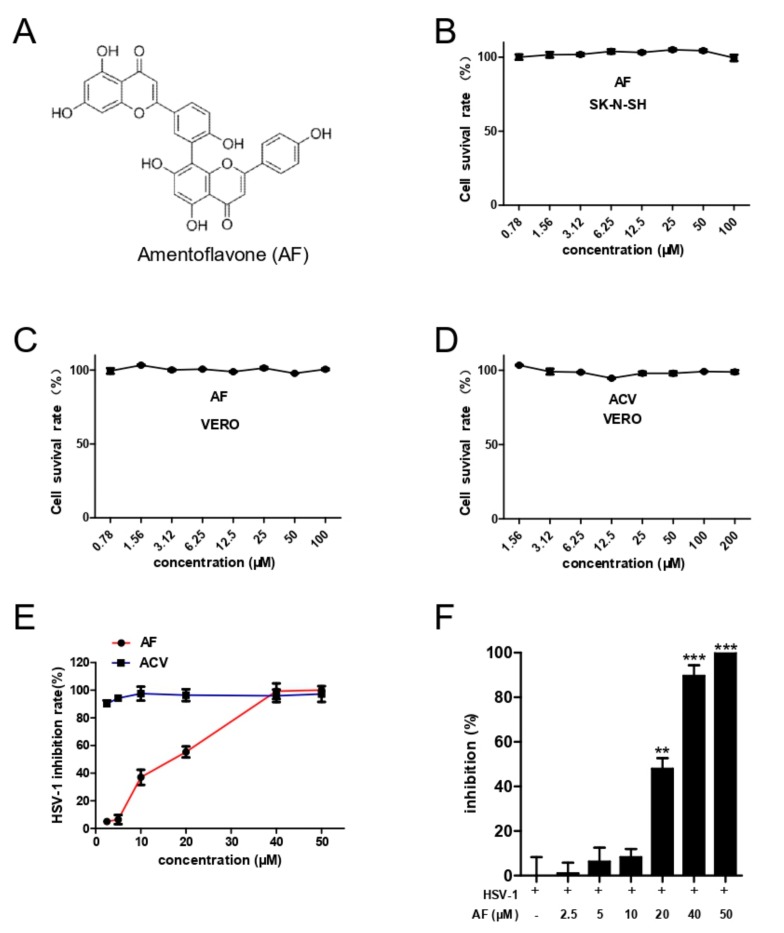
Toxicities and antiviral activities of amentoflavone. (**A**) Chemical structure of amentoflavone. (**B**–**D**) Cytotoxicities of ACV and amentoflavone. SK-N-SH and Vero cells were treated with different concentrations of amentoflavone or ACV for 72 h and cell viability was calculated by CCK8 assay. (**E**,**F**) Anti-HSV-1 activity of amentoflavone. Vero cells were infected with HSV-1 (MOI = 0.1) in the presence of amentoflavone or ACV for 24 h and CPE assay was used to estimate the inhibitory effect (**E**). The cells infected with HSV-1 (MOI = 0.1) were treated with amentoflavone or DMSO (control group) for 72 h, and the inhibitory effect of amentoflavone was estimated by plaque assay (**F**). Data are mean ± SD (*n* = 3). ** *p* < 0.01; *** *p* < 0.001 versus HSV-1-treated group.

Numerous potent anti-HSV-1 compounds are derived from natural plants [15,16]. Amentoflavone (AF; Figure 1A), a polyphenol compound that extensively exists in *Biophytum sensitivum* and other plants including edible *Garcinia Species* and *Juniperus communis L* [17,18,19], displays many pharmacological properties, including anti-inflammatory, antioxidative, antitumor, and neuroprotective activity [20,21,22,23,24]. In addition, amentoflavone has been found to be a broad spectrum antivirus compound against a series of viruses [25].

In this study, we evaluated whether amentoflavone can inhibit HSV-1 infection and revealed the mechanism thereof. The anti-ACV-resistant virus activity of amentoflavone was also investigated.

## 2. Materials and Methods

### 2.1. Cells and Viruses

Vero cell line (ATCC, USA) was cultured in Dulbecco’s modified Eagle’s medium (DMEM; 8118305, GIBCO/Thermo Fisher Scientific, USA) with 10% fetal bovine serum (FND500, ExCell Bio, Shanghai, China). The neuroblastoma cell line SK-N-SH (ATCC, HTB-11, American Type Culture Collection, Manassas, VA, USA) was propagated in Eagle’s minimal essential medium (MEM; GIBCO /Thermo Fisher Scientific, USA) supplemented with 10% FBS. In our previous studies, HSV-1 infection induced the biphasic F-actin dynamics in SK-N-SH cells [26]. In this study, the SK-N-SH cells were used to study the effect of amentoflavone on F-actin. HSV-1 strain F (ATCC, USA), initially obtained from Hong Kong University, was propagated in Vero cells and stored at −80 °C until use. HSV-1/Blue, a TK mutant derived from HSV-1 (KOS) and two acyclovir-resistant clinical HSV-1 strains HSV-1/106 and HSV-1/153 were kind gifts from Tao Peng, State Key Laboratory of Respiratory Disease, Guangzhou Institutes of Biomedicine and Health, Chinese Academy of Sciences. GFP-HSV-1, expressing a GFP-tagged viral protein Us11, was used to evaluate viral nuclear transport [27]. HSV-1 Us11 is a multifunctional late protein which can regulate the accumulation of RNA species and facilitate HSV-1 replication [28]. 

### 2.2. Compounds, Antibodies, and Reagents

Amentoflavone was purchased from Selleck (S3833, Houston, TX, USA) and dissolved in Dimethyl Sulphoxide with a concentration of 20 mM. Acyclovir (ACV) was purchased from Sigma-Aldrich (St. Louis, MO, USA). Cytochalasin B (CB) was purchased from Sigma-Aldrich (14930-96-2, St. Louis, MO, USA). Antibodies, including anti-ICP0 (ab6513), anti-VP5 (ab6508), and anti-gD (ab6507), were purchased from Abcam (Cambridge, UK), anti-VP22 was purchased from Santa Cruz Biotechnology (Santa Cruz, CA, USA), anti-cofilin (5175S), anti-p-cofilin (3313S), anti-GAPDH (5174S), and anti-Acetyl-α-Tubulin (5335S) were obtained from Cell Signaling Technology (Danvers, MA, USA). TRIzol Reagent was brought from TIANGEN (Beijing, China).

### 2.3. Cytotoxicity and Antiviral Activity Assay

1 × 10^4^ Vero cells or SK-N-SH cells were seeded in 96-well plates and incubated at 37 °C, 5% CO_2_ overnight. The supernatant was then removed and new medium with amentoflavone or acyclovir was added. After 72 h, the CCK8 reagent (10 μL/well) was added for 2 h to record the OD value by enzyme immunoassay reader at 490 nm. The 50% cytotoxic concentration (CC_50_) was calculated accordingly [29].

Viral titration was used to determine cytopathic effects (CPEs) in Vero cells and the 50% tissue culture infectious dose (TCID50) was calculated [30]. Subsequently, the TCID50/mL was converted into plaque-forming units (PFU)/mL [31]. The assays were conducted as described in our previous studies [32,33]. Briefly, 1 × 10^4^ or 1.5 × 10^5^ Vero cells were seeded in 96-well or 24-well plates to perform CCK8 assay or plaque assay, respectively. The cells were infected with HSV-1/F, HSV-1/106, HSV-1/153 or HSV-1/Blue (MOI = 0.1) for 2 h, and the culture medium was then replaced with new medium containing amentoflavone at 37 °C, 5% CO_2_. After 72 h, the OD value of the cells in 96-well plates was detected by CCK8 assay. The cells in 24-well plates were fixed by paraformaldehyde for 0.5 h, and strained with crystal violet for 0.5 h. Finally, the number of plaques was counted to calculate the inhibition rate of virus infection as described previously [32]. The 50% effective concentration (EC_50_) was also calculated as described previously [33].

### 2.4. Quantitative Real-Time PCR (qRT-PCR)

The cells were treated on the basis of diverse experimental requirements. Total RNA was extracted using TRIzol and the RNA concentration was measured at 260/280 nm using a NanoPhotometer P330 spectrophotometer (IMPLEN, Munich, Germany). One μg RNA was reverse transcribed into cDNA using a reagent kit (PrimeScript RT reagent Kit, Takara, Shiga, Japan). Afterwards, the mRNA expression levels of viral genes were analyzed using Bio-Rad CFX96 real-time PCR system (Bio-Rad) according to previous studies [26]. The expression level of 18SrRNA was used as a reference. The primer sequences were as follows: HSV-1 *UL54* F (5′-TGGCGGACATTAAGGACATTG-3′), *UL54* R (5′-TGGCCGTCAACTCGCAGA-3′), *UL52* F (5′-GACCGACGGGTGCGTTATT-3′), *UL52* R (5′-GAAGGAGTCGCCATTTAGCC-3′), *UL27* F (5′-GCCTTCTTCGCCTTTCGC-3′), *UL27* R (5′-CGCTCGTGCCCTTCTTCTT-3′), *ICP0* F (5′-CCCACTATCAGGTACACCAGCTT-3′), *ICP0* R (5′-CTGCGCTGCGACACCTT-3′), *ICP4* F (5′-CGACACGGATCCACGACCC-3′), *ICP4* R (5′-GATCCCCCTCCCGCGCTTCGTCCG-3′), and *18SrRNA* F (5′-CATGGTGACCACGGGTGAC-3′), *18S rRNA* R (5′-TTCCTTGGATGTGGTAGCCG-3′). For viral nuclear transport assay, the DNA copy number of viral gene *UL52* and *UL27* was detected [13]. Total DNA was extracted using TIANamp Virus DNA/RNA Kit (TIANGEN, Beijing, China) and targeted genes were analyzed by qRT-PCR. 

### 2.5. Nuclear and Cytoplasmic Protein Extraction

Vero cells infected with HSV-1 (MOI = 10) for 2 h at 4 °C were treated with AF (50 μM) for another 2 h at 37 °C. The cells were then harvested and swelled in Buffer A (10mM Hepes, 1 mM MgCl_2_, 10 mM KCl, 0.5 mM DTT) for 15 min on ice. After discarding the supernatant, the cells were lysed with Buffer A containing 10% NP-40, 1mM PMSF, and subjected to a vortex for 10 s. After centrifugation, the supernatant was harvested as cytoplasmic protein, and the cell pellet was resuspended in Buffer B (20 mM Hepes/PH7.9, 25% glycerine, 0.42 mM NaCl, 0.2 mM EDTA, 0.5 mM DTT) containing PMSF to lyse for 30 min. The supernatant was harvested as nuclear protein after centrifugation.

### 2.6. Western Blot Assay

Vero cells or SK-N-SH cells were infected with HSV-1 (MOI = 1) in the presence or absence of amentoflavone. Cell lysates were collected at various time points using SDS buffer (Beyotime, ShangHai, China), and proteins were separated by 10% gradient SDS-PAGE, transferred to polyvinylidene fluoride (PVDF) membrane (Millipore), and were then blocked with 5% nonfat milk for 1 h at room temperature. Targeted proteins were incubated with primary antibodies overnight at 4 °C and with the secondary antibodies for 1 h at room temperature. Finally, those target proteins were detected by ECL solutions. The band intensity of each protein was calculated using Quantify One software (Bio-Rad, Hercules, CA, USA) and was normalized to that of GAPDH.

### 2.7. Immunofluorescence Assay

Vero cells or SK-N-SH cells infected with HSV-1 in the presence of amentoflavone (50 μM) were washed with phosphate-buffered saline (PBS) for three times, fixed with 4% paraformaldehyde for 20 min, permeabilized with 0.1% Triton X-100 for 5 min, and blocked with 5% bovine serum albumin for 1 h. Subsequently, the samples were incubated with anti-gD or anti-VP5 antibodies overnight at 4 °C. Next, the samples were incubated with Alexa Fluor 488 (green)-labelled secondary antibody (life technologies) for 1 h. In addition, cell nucleus was stained with DAPI (blue) (C1006, Beyotime, ShangHai, China) and cell actin filaments were stained by FITC-labelled phalloidin (red). Finally, fluorescent images were obtained using a confocal laser scan microscope (LSM 510 meta; Zeiss, Jena, Germany).

### 2.8. Flow Cytometry Assay

SK-N-SH cells infected with or without HSV-1 were treated with amentoflavone (50 μM) or cytochalasin B (CB, 20 μM). After 1 h at 37 °C, the cells were washed with PBS, fixed with 4% paraformaldehyde for 5 min, and permeabilized with 0.1% Triton X-100 for 5 min. Next, the samples were stained with 5% TRITC-Phalloidin (YEASEN, 40734ES75) for 40 min at 37 °C. Finally, the fluorescence was analyzed with a flow cytometer (Becton Dickinson, CA, USA).

### 2.9. Luciferase Reporter Gene Assay

The effect of amentoflavone on the promoter activity of viral immedi ate early gene was analyzed using a dual luciferase assay [27]. Briefly, the promoter sequence of viral immediate early gene *α0* and *α4* was cloned into the luciferase reporter plasmid pGL4.12 [luc2p] (Promega, Madison, WI, USA) according to the manufacturer’s instructions. HSV-1 virion protein 16 (VP16) is a crucial protein involved in the assembly of a transactivation complex binding to the promoters of viral *α0* and *α4*. Therefore, exogenous expression of VP16 was used as a positive control and the protein coding sequence of VP16 was cloned into the expression plasmid pcDNA3.1 (pcDNA). Vero cells were transfected with pcDNA3.1(+)-VP16 plasmid (pcDNA-VP16) (250 ng/well) in combination with the pGL4.12 [luc2p]-*α0* promoter (p-GL*α0*) plasmid (250 ng/well) or pGL4.12 [luc2p]-*α4* promoter (p-GL*α4*) plasmid (250 ng/well) using a jetPRIME® kit (PT-114-15; Polyplus Transfection, France), respectively. The pRL-TK plasmid (5 ng/well) was transfected as an internal reference. After transfection, the cells were treated with amentoflavone (50 μM). Dual-Luciferase®Reporter assay was performed using a GloMax 20/20 GloMax20/20 instrument (Promega, USA).

### 2.10. Statistical Analysis

Data are presented as mean ± SD. Data were analyzed by one-way analysis of variance or Student’s *t* test as appropriate, and the level of significance was set at *p* < 0.05 (*), *p* < 0.01 (**), or *p* < 0.001 (***).

## 3. Results

### 3.1. Cytotoxicity and Comprehensive Antiviral Activity of Amentoflavone

The cytotoxicity of amentoflavone and ACV on Vero cells and SK-N-SH cells was detected by CCK8 assay (Figure 1B–D), and the CC_50_ values are shown in Table 1. In addition, the antiviral activity of amentoflavone was estimated by CPE and plaque assay, respectively, which showed that amentoflavone inhibited HSV-1 infection in a dose-dependent manner (from 2.5 to 50 μM) (Figure 1E,F). As expected, the antiviral drug ACV showed excellent antiviral effect at a low concentration of 2.5 μM. Finally, plaque assay was performed to determine the EC_50_ values (Table 2). Taken together, amentoflavone displays strong anti-HSV-1 activity at high concentration.

### 3.2. Amentoflavone Inhibits HSV-1 Gene and Protein Expression

**Figure 2:**
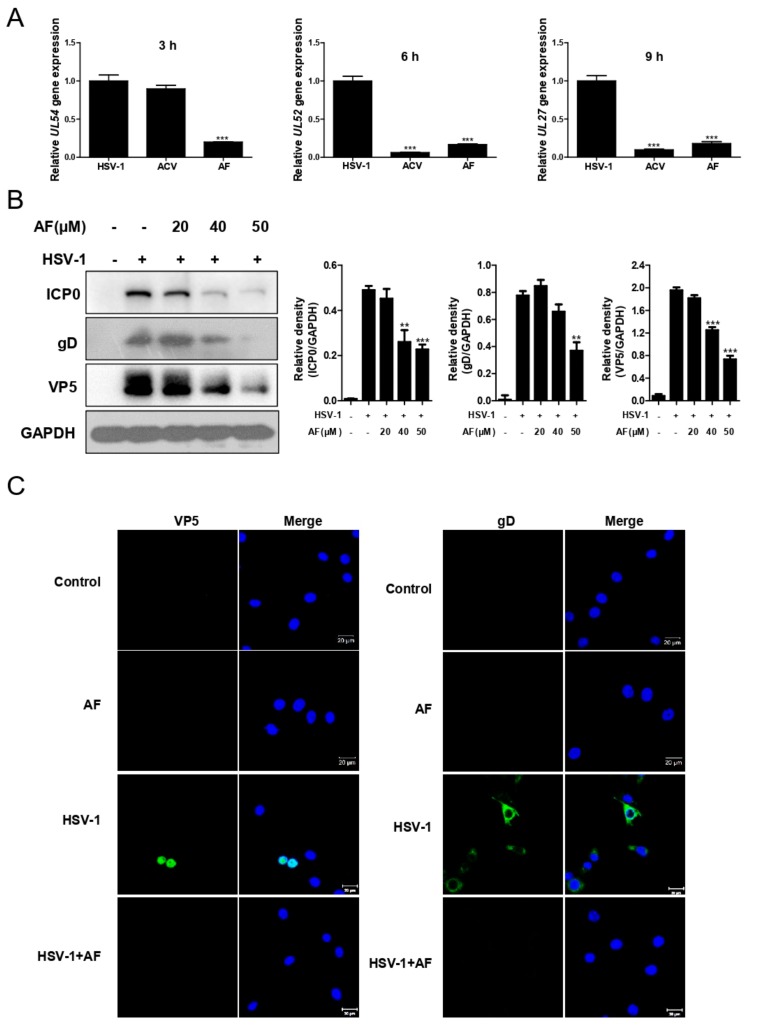
Amentoflavone inhibits viral gene and protein expression. (**A**) Vero cells were infected with HSV-1 (MOI = 1) in the presence of ACV (50 μM), amentoflavone (50 μM) or DMSO (control group). At different time points (3, 6, 9 h p.i.), total RNA samples were extracted and the mRNA expression levels of *UL54* (immediate early gene), *UL52* (early gene), and *UL27* (late gene) were detected by qRT-PCR. The mRNA expression was normalized to 18s RNA. Data are mean ± SD (*n* = 3). *** *p* < 0.001 versus HSV-1-treated group. (**B**) Amentoflavone inhibited viral protein expression. The cells were infected with HSV-1 for 3 h and the representative protein level of viral immediate early protein ICP0 was detected by Western Blot. For viral late protein gD and VP5, the cells were infected with HSV-1 for 9 h. Densitometric analysis for all western blot bands was shown. GAPDH served as a loading control. Data are mean ± SD (*n* = 3). ** *p* < 0.01; *** *p* < 0.001 versus HSV-1-treated group. (**C**) The cells were infected with HSV-1 (MOI = 1) for 9 h in the presence of amentoflavone (50 μM) or DMSO (control group), and were then fixed, stained with primary antibody against VP5 or gD (green). Nucleus was stained with DAPI (Blue). Images were recorded by a confocal LSM.

Next, we evaluated the effect of amentoflavone on HSV-1 gene and protein expression. Indeed, the mRNA expression of several representative viral genes, including *UL54* (viral immediate early gene, IE), *UL52* (early gene) and *UL27* (late gene), was significantly reduced by amentoflavone (Figure 2A). Consistently, amentoflavone markedly inhibited protein levels of viral immediate early protein ICP0, late protein gD and VP5 (Figure 2B). Immunofluorescence assay further reinforced the conclusion that amentoflavone reduces the expression of viral proteins (Figure 2C).

### 3.3. Amentoflavone Inhibits ACV-Resistant Strains Infection

**Figure 3:**
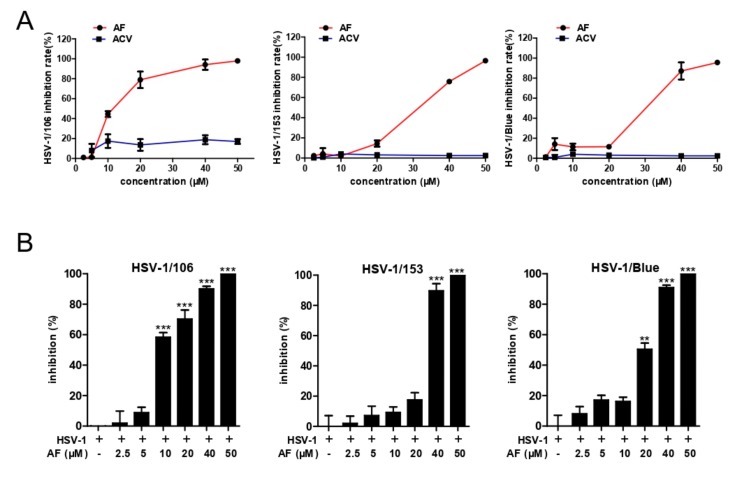
Amentoflavone inhibits infection by ACV-resistant HSV-1 strains. (**A**) Vero cells were infected with HSV-1/106, HSV-1/153, and HSV-1/Blue (MOI = 0.1) in the presence of amentoflavone or ACV for 72 h. The antiviral effect was assessed by CCK8 assay. (**B**) Vero cells were infected with HSV-1/106, HSV-1/153, and HSV-1/Blue (MOI = 0.1) for 2 h. The viruses were then removed and cover fluid with amentoflavone was added. After 72 h, the cells were fixed and strained with crystal violet dye. The plaque numbers were counted to calculated the inhibitory effect. Data are mean ± SD (*n* = 3). ** *p* < 0.01, *** *p* < 0.001 versus HSV-1-treated group.

ACV mainly acts as a substrate of HSV Thymidine kinase (TK) and inhibits viral DNA replication, and ACV-resistant HSV clinical isolates are TK-negative, TK-low-producer mutants, and TK-altered mutants [34]. In this study, we also assessed whether amentoflavone had antiviral activity against ACV-resistant strains, including HSV-1/Blue, a TK mutant derived from HSV-1, and two clinical HSV-1 strains HSV-1/106 and HSV-1/153 [35]. As shown in Figure 3A, no antiviral activity of ACV toward those viruses was observed even at a high concentration (50 μM). In contrast, amentoflavone significantly inhibited HSV-1/106 strain at the concentration of 20 μM and inhibited HSV-1/153 strain and HSV-1/blue strain at the concentration of 40 μM, respectively. In addition, plaque assay showed that amentoflavone completely inhibited all ACV-resistant strains at 50 μM (Figure 3B), consistent with CPE results. The EC_50_ values are shown in Table 2.

**Figure 4:**
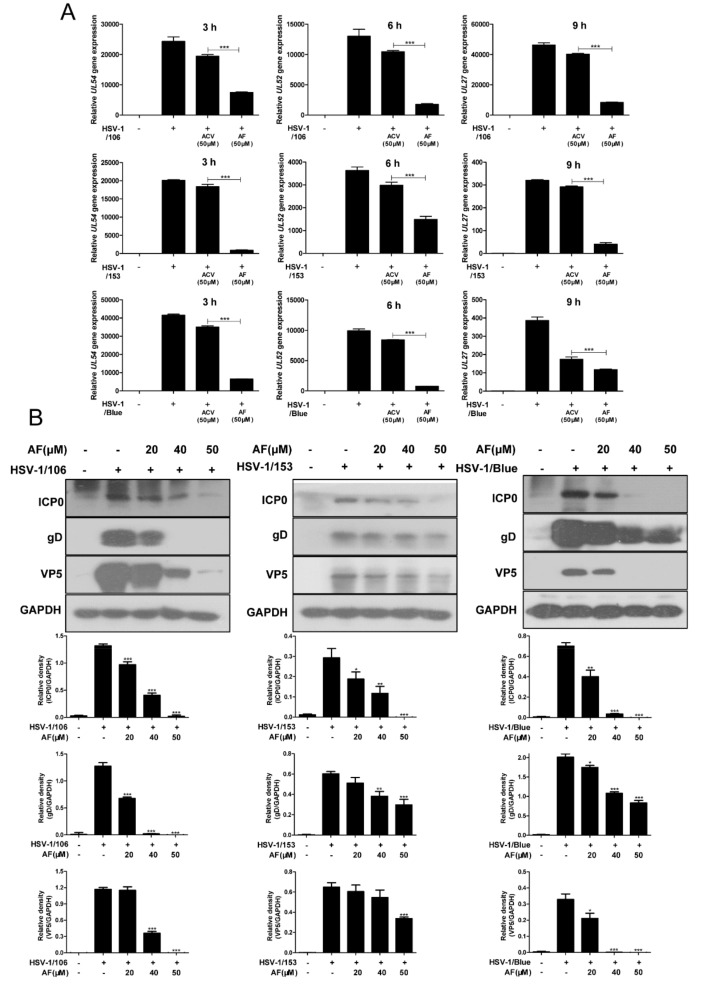
Amentoflavone affects the gene and protein expression of ACV-resistant viruses. (**A**) Vero cells were infected with HSV-1/106, HSV-1/153, and HSV-1/Blue (MOI = 1) in the presence of ACV (50 μM), amentoflavone (50 μM), or DMSO (control group). Total RNA samples were extracted to detect the expression levels of *UL54*, *UL52*, and *UL27* at 3, 6, and 9 h p.i., respectively. Data are mean ± SD (*n* = 3). *** *p* < 0.001. (**B**) The expression of various viral proteins was detected by Western Blot. The cells infected with HSV-1 were treated with amentoflavone or DMSO (control group) for 3 h and total proteins were subjected to western blot for ICP0 analysis. For viral late protein gD and VP5, the cells were infected with HSV-1 for 9 h in the presence of amentoflavone or DMSO. Different protein concentrations for the different virus samples were used for Western blot. Data are mean ± SD (*n* = 3). * *p* < 0.05; ** *p* < 0.01; *** *p* < 0.001 versus HSV-1-treated group.

Furthermore, we evaluated the inhibitory effect of amentoflavone on the gene and protein expression of all three ACV-resistant viruses. As expected, amentoflavone almost completely suppressed viral gene production (*UL54*, *UL52*, and *UL27*) (Figure 4A). Western Blot assay also clearly demonstrated that amentoflavone exhibited a dose-dependent inhibitory effect on viral proteins, as illustrated by protein ICP0, gD, and VP5 (Figure 4B). The differences in viral protein expression among three ACV-resistant viruses were mainly attributed to the unequal concentrations of total proteins used for western blot assay. All these results indicated that amentoflavone has the ability to inhibit ACV-resistant virus.

### 3.4. Amentoflavone Reduces the Nuclear Import of HSV-1

**Figure 5:**
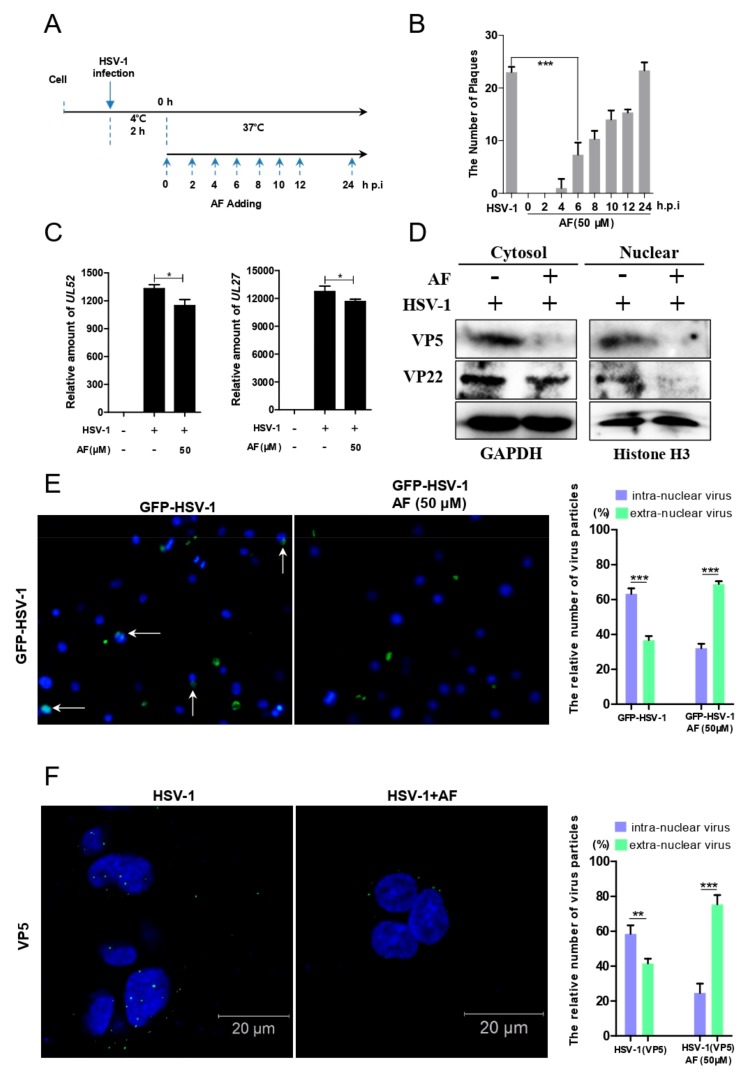
Amentoflavone impairs the viral nuclear transport process. (**A**) Simple diagram of the time-addition assay. (**B**) Vero cells were treated with HSV-1 (MOI = 0.1) at 4 °C to adsorb. After 2 h, the cells were washed with PBS to remove the virions and were subsequently incubated at 37 °C. Then, amentoflavone was added at different time points. After 72 h, the cells were fixed and strained with crystal violet dye. Data are mean ± SD (*n* = 3), *** *p* < 0.001. (**C**) The cells were challenged with HSV-1 (MOI = 10) at 4 °C for 2 h, and were treated with amentoflavone for another 2 h. The total DNA was extracted, and the expression level of UL52 and UL27 was detected. Data are mean ± SD (*n* = 3), * *p* < 0.05. (**D**) The cells infected with HSV-1 (MOI = 10) for 2 h at 4 °C were treated with amentoflavone (50 μM) or DMSO (control group) for another 2 h at 37 °C, and the total proteins in the cytoplasm and nucleoplasm were extracted. The protein levels of viral proteins VP5 and VP22 were analyzed by western blot assay. (**E**) Vero cells were challenged with GFP-HSV-1 (MOI = 10) at 4 °C for 2 h, and were treated with amentoflavone or DMSO (control group) for another 2 h at 37 °C. The cells were then fixed and the immunofluorescence images were acquired by a Nikon microscope. In addition, the number of intra/extranuclear virus particles was counted in 100 fields. White arrows indicated the GFP-labeled HSV-1 virion within nucleus. Data are mean ± SD (*n* = 3), *** *p* < 0.001. (**F**) SK-NSH cells were infected with HSV-1 for 2 h at 4 °C and were treated with amentoflavone or DMSO (control group) for another 2 h at 37 °C. The cells were then fixed and stained with anit-VP5 primary antibody (green) and DAPI (blue). The immunofluorescence images were acquired by a confocal LSM. Data are mean ± SD (*n* = 3), ** *p* < 0.01, *** *p* < 0.001.

To determine which step of HSV-1 infection amentoflavone affected, time-of drug-addition assay was performed. As shown in Figure 5A,B, the number of plaques was significantly reduced when amentoflavone was added at the time point 0–4 h.p.i, suggesting that amentoflavone mainly affected HSV-1 early infection. The early steps of HSV-1 infection include viral attachment to host cell membrane, penetration, and subsequent transportation to nucleus. To evaluate whether amentoflavone affected viral nuclear transport, Vero cells were infected with HSV-1 for 2 h in the absence or presence of amentoflavone and total DNA was extracted to analyze the DNA copy number of viral gene *UL52* and *UL27* as described in our previous work [13]. We found that the relative amount of *UL52* and *UL27* in the amentoflavone-treated group was lower than in the HSV-1-treated group, suggesting that viral entry was affected by amentoflavone (Figure 5C). To further confirm the above result, we extracted nuclear and cytoplasmic protein at 2 h.p.i, respectively. As showed in Figure 5D, both nuclear and cytoplasmic viral protein VP5 and VP22 was largely reduced by amentoflavone treatment. Next, GFP-HSV-1, expressing a GFP-tagged viral protein, US11, was used to further assess the effect of amentoflavone on viral nuclear transport process (Figure 5E). GFP-labelled viral particles reached the nucleus at 2 h.p.i in control group, whereas most virions were blocked in the cytoplasm in the presence of amentoflavone, implying impaired nuclear transport of HSV-1. Finally, we performed immunofluorescence assay using viral tegument protein VP5 as an indication of HSV-1 particles [36]. The viruses were mostly being transferred to the nucleus in control group. However, treatment with AF significantly reduced the number of viral particles and only a few viral particles docked at the nucleus (Figure 5F). Taken together, amentoflavone significantly reduced the nuclear transport of viral particles.

**Figure 6:**
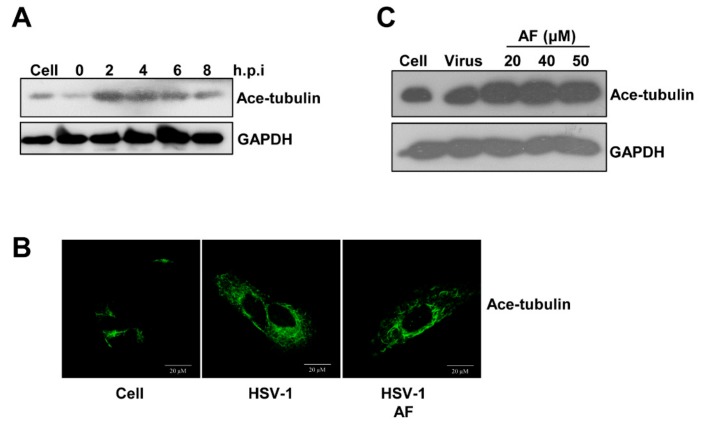
Amentoflavone does not affect microtubule organization. (**A**) SK-N-SH cells seeded in 30 mm^2^ dish were infected with HSV-1 (MOI = 10) at 4 °C for 2 h, and were then incubated at 37 °C for different time points. Total proteins were extracted to detect the level of acetylated tubulin. (**B**) The SK-N-SH cells infected with HSV-1 were treated with amentoflavone or DMSO (cell and HSV-1 group) for 2 h at 37 °C. The cells were then fixed and stained with anti- ace-tubulin primary antibody. (**C**) SK-N-SH cells seeded in a 30 mm^2^ dish were infected with HSV-1 (MOI = 10) at 4 °C for 2 h, and then incubated at 37 °C in the presence of amentoflavone or DMSO (HSV-1 control group) for 2 h at 37 °C. Total proteins were extracted to detect the level of acetylated tubulin.

Viral intracellular transportation relies on the dynamic regulation of the cell’s cytoskeleton, including F-actin and the microtubule system. In the previous study, we have showed that early infection of HSV-1 mobilizes tubulin and F-actin reorganization to facilitate viral entry and nuclear transport [14,26]. Biphasic F-actin depolymerization and polymerization promotes HSV-1 entry and penetration, whereas acetylation of tubulin facilitates nuclear transportation of viral particles. Therefore, we first analyzed whether the reorganization of microtubule was involved in the antiviral effect of amentoflavone. Indeed, HSV-1 infection increased acetylated-tubulin levels in neuronal cells detected by western blot assay (Figure 6A). However, such enhanced acetylated tubulin remained unchanged when amentoflavone was added (Figure 6B,C), suggesting the involvement of other cytoskeleton components. 

**Figure 7:**
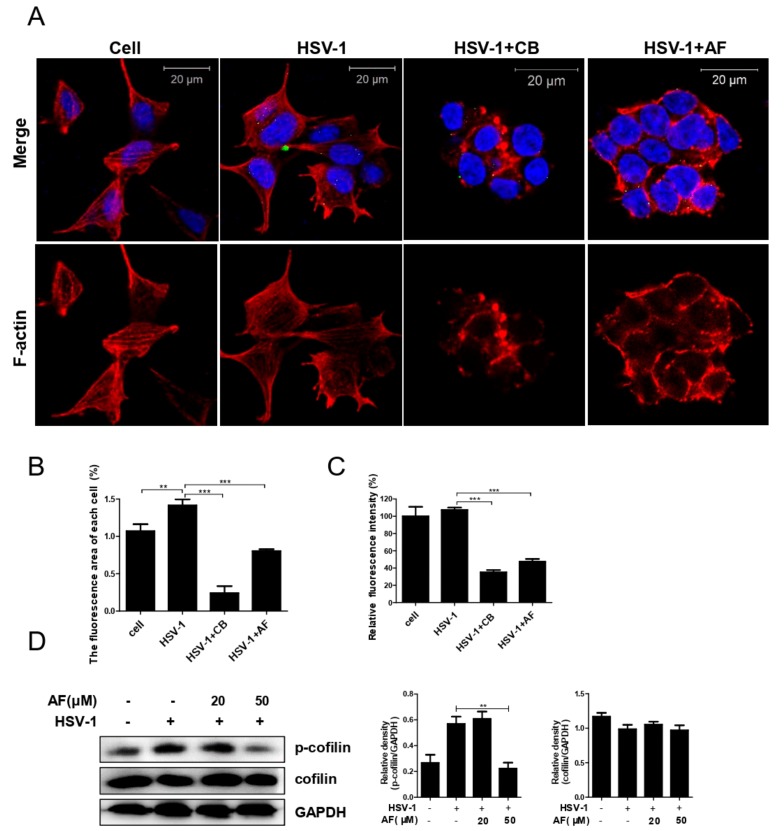
Amentoflavone disturbs cofilin-mediated F-actin assembly. SK-N-SH cells seeded in confocal dishes or six-well plates were infected with HSV-1 (MOI = 10) at 4 °C for 2 h, and were treated with cytochalasin B (CB, 20 μM), amentoflavone (50 μM), or DMSO (cell and HSV-1 group) for 1 h at 37 °C. (**A**,**B**) The cells were then stained with anti-VP5 primary antibody (green), TRITC-phalloidin (F-actin, red), and DAPI (nucleus, blue). (**B**) Image J software was used to analyze the percent of F-actin fluorescence area of each cell in images (50 cells). Data are mean ± SD (*n* = 3). ** *p* < 0.01, *** *p* < 0.001. (**C**) F-actin was stained with TRITC-phalloidin and was analyzed by flow cytometry (Becton Dickinson). Data are mean ± SD (*n* = 3). *** *p* < 0.001. (**D**) After HSV-1 attachment at 4 °C for 2 h, SK-N-SH cells were shifted to 37 °C for 1 h in the presence of amentoflavone or DMSO. The protein levels of total or phosphorylated cofilin were detected by western blot. Data are mean ± SD (*n* = 3). ** *p* < 0.01.

Considering that cofilin-mediated F-actin polymerization promotes HSV-1 binding and entry, whereas F-actin depolymerization facilitates viral penetration and subsequent nuclear transportation [14], we thus analyzed the possible effect of amentoflavone on F-actin-mediated HSV-1 early infection. As shown in Figure 7A, HSV-1 infection clearly induced the formation of lamellipodia and filopodia (different forms of accumulated F-actin), or the formation of cortical actin in accordance with our previous works [13,26]. In contrast, cytochalasin B (CB), which induces the depolymerization of existing actin filaments, dramatically disturbed F-actin and reduced viral particles docked in cytoplasm. In addition, AF treatment exhibited a similar effect as CB that substantially impaired the reorganization of F-actin and viral infection (Figure 7A,B). Moreover, AF-mediated F-actin remodeling was further confirmed by flow cytometry analysis (Figure 7C). Similarly, both CB and AF induced the depolymerization of HSV-1-induced F-actin as the fluorescence intensity of F-actin was largely reduced by CB and AF. These results clearly indicated that amentofalvone perturbed HSV-1-induced F-actin dynamics. Furthermore, we analyzed the activity of cofilin, a key regulator for F-actin polymerization, and found that amentoflavone reduced the phosphorylation level of cofilin during early viral infection (Figure 7D). Considering these results together with our previous report [14], it is possible that inhibition of cofilin by amentoflavone may thereby interrupt viral membrane docking and subsequent intracellular transportation. Taken together, amentoflavone influenced cofilin-mediated F-actin assembly and reduced the nuclear import of HSV-1.

### 3.5. Amentoflavone Reduces Immediate Early Gene Promoter Activity

**Figure 8:**
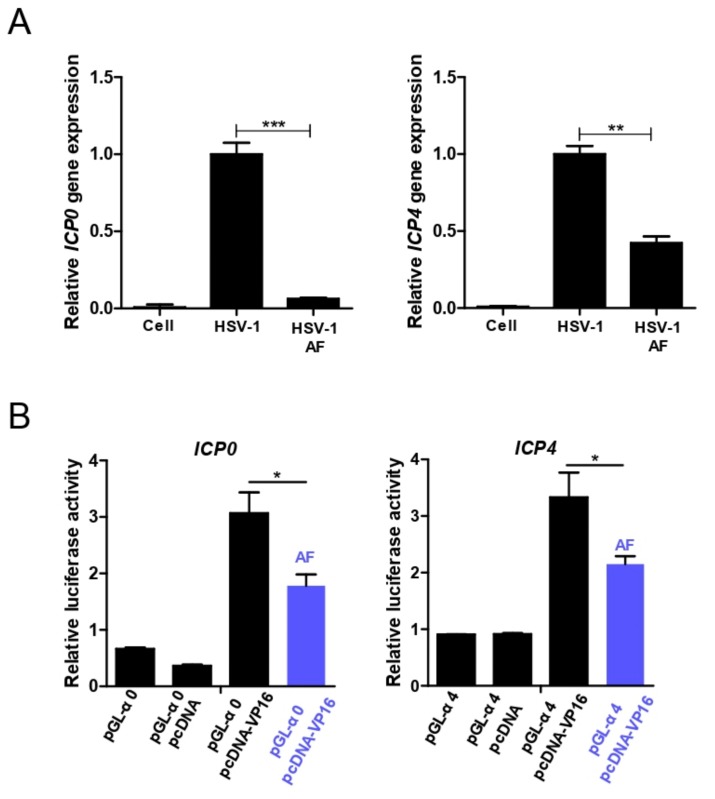
Amentoflavone inhibits the promoter activity of viral immediate early gene. (**A**) Vero cells were infected with HSV-1 (MOI = 1) in the presence of amentoflavone (50 μM) or DMSO for 3 h and the expression of ICP0 and ICP4 was detected by qRT-PCR, respectively. Data are mean ± SD (*n* = 3). ** *p* < 0.01; *** *p* < 0.001. (**B**) Vero cells seeded in 48-well plates were transfected with indicated plasmid combinations for 24 h, the cell lysates were then subjected to luciferase activity assays. The cells only transfected with pGL-α0 plasmids (pGL-α4 plasmids) or cotransfected with pGL-α0 and pcDNA plasmids (pGL-α4 and pcDNA plasmids) were treated as the negative control. Data are mean ± SD (*n* = 3). * *p* < 0.05.

Our above results clearly demonstrated that treatment of infected cells with amentoflavone almost completely inhibited HSV-1 infection at 4 h.p.i, and substantially decreased the expression of early viral genes (Figure 2A and Figure 4A); therefore, we speculated that amentoflavone might affect the activity of the immediate early gene promoter. Firstly, we tested whether amentoflavone broadly inhibited HSV-1 immediate early gene expression and found that the expression of *ICP0*, *ICP4*, and *UL54* was decreased in the presence of amentoflavone (Figure 8A). Then, we constructed the luciferase reporter gene under the control of the promoter of the *ICP0* and *ICP4* genes and performed a dual luminal assay to evaluate the activity of amentoflavone (Figure 8B). HSV-1 tegument protein VP16 promotes the formation of a transactivation complex, which binds to the promoters of immediate early genes to initiate their gene expression [37]. Indeed, overexpression of VP16 significantly increased luciferase activity in the pcDNA3.1(+)-VP16 plasmid and pGL-α0-transfected group. We also found that such enhanced VP16-mediated luciferase activity was suppressed by amentoflavone (Figure 8B). These results confirmed that amentoflavone suppressed the immediate early gene expression partly by inhibiting its promoter activity.

## 4. Discussion

There is a clear need for the development of new antiviral agents against gradually emerging ACV-resistant HSV strains. In this study, we showed that amentoflavone had antiviral activity against HSV and ACV-resistant strains. Mechanically, amentoflavone affected cofilin-mediated F-actin remodeling and reduced viral nuclear transportation to suppress HSV-1 early infection. 

Amentoflavone has been showed to inhibit the infection of various viruses, such as herpes viruses, influenza A and B viruses, and coxsackie virus B3 [23,38]. For instance, amentoflavone decreases coxsackievirus B3 replication by inhibiting fatty acid synthase [25]. However, the detailed mechanisms about how amentoflavone inhibits herpes and influenza virus are largely unknown. In our work, we found that amentoflavone not only inhibited normal HSV virus, but also significantly suppressed ACV-resistant strains, including HSV-1/Blue, HSV-1/106, and HSV-1/153. We further investigated the anti-HSV-1 mechanism and showed that amentoflavone affected viral early infection events, such as viral nuclear translocation and viral immediate early gene expression.

Time-point-addition experiments revealed that amentoflavone mainly affected HSV-1 early infection (Figure 5B). Early infection events consist of viral binding, penetration, and intracellular nuclear transportation, all of which rely on the reorganization of the cell’s cytoskeleton, including actin filaments and microtubules [7,8]. The microtubule system plays a central role in cell division and spatial organization of the cell cytoplasm and viruses are able to hijack the microtubule transport system for intracellular transportation of virion/viral genomic material to the sites of replication, assembly, and egress. We have also demonstrated that HSV-1 nuclear translocation requires the rearrangement of tubulin [36]. However, both western blotting assay and immunofluorescence experiments showed that the microtubule system was not influenced by amentoflavone, as indicated by the unchanged level of acetylated tubulin (Figure 6). Cortical actin acts as an obstacle to pathogen entry or egress and, as a consequence, different viruses have evolved to utilize various strategies that subvert the actin cytoskeleton to facilitate their infections [9,10,12]. For instance, HSV-1 and HIV modulate cofilin-mediated actin biphasic polymerization and depolymerization to promote viral internalization and penetration via EGFR-PI3K signaling and CXCR4-PAK signaling, respectively [11,14]. The actin regulator cofilin is the specific cellular machinery usurped by virus infection [12]. In addition, F-actin dynamic-disrupting drugs strongly reduce the transport of HSV-1 [26,39]. Similarly, in our work, we found that amentoflavone reduced F-actin assembly (Figure 7). Amentoflavone also inhibited the phosphorylation of cofilin, the key regulator of F-actin dynamics, at 1 h.p.i (Figure 7D). Whether the upstream signaling of cofilin, such as EGFR-PI3K-LIMK, was affected by amentoflavone remains to be further investigated.

Finally, we found that amentoflavone remarkably inhibited immediate early gene expression of both ordinary HSV strain and ACV-resistant strains via attenuating promoter activity. The expression of immediate-early genes requires the viral VP16-HCF-1-Oct-1 complex to stimulate its promoter activity independently of DNA replication [40]. It will be interesting to verify whether amentoflavone interferes with the interaction among each component of this complex. Alternatively, other activities of amentoflavone may also contribute to its antiviral effect. For instance, HSV-1 infection triggers severe inflammatory responses to damaged cells, whereas amentoflavone can significantly suppress NF-κB-mediated inflammation [21]. Autophagy plays a crucial role in antiviral innate immunity and HSV-1-encoded neurovirulence protein ICP34.5 directly binds to autophagy regulator Beclin 1 to inhibit autophagy [41]. Importantly, amentoflavone has a positive effect on the induction of autophagy [42], which may in turn stimulate the antiviral activity of autophagy. 

In summary, our results demonstrated that amentoflavone inhibited common HSV-1 F strain and ACV-resistant strains though influencing viral nuclear transport process and immediate early gene promoter activity. The antiviral effects of amentoflavone, especially for ACV-resistant strains, should be further evaluated in animal studies.

## Figures and Tables

**Table 1:** The cytotoxicity of amentoflavone and ACV on Vero and SK-N-SH cells.

Cell/Compounds	CC_50_ (μM)
Vero/ACV	>100
Vero/AF	>100
SK-N-SH/AF	>100

CC_50_, 50% cellular cytotoxicity concentration; Data are mean ± SD (*n* = 3).

**Table 2:** The antiviral activity of amentoflavone on four HSV-1 strains.

Virus (Vero)	EC_50_ (μM)
HSV-1	22.13 ± 0.38
HSV-1/106	11.11 ± 0.71
HSV-1/153	28.22 ± 2.51
HSV-1/Blue	25.71 ± 3.97

EC_50_, the 50% effective concentration; Data are mean ± SD (*n* = 3).
